# Rehabilitation during early postoperative period following total knee arthroplasty using single-joint hybrid assistive limb as new therapy device: a randomized, controlled clinical pilot study

**DOI:** 10.1007/s00402-021-04245-9

**Published:** 2021-11-16

**Authors:** Silvia J. Mrotzek, Shahir Ahmadi, Alexander von Glinski, Alexis Brinkemper, Mirko Aach, Thomas A. Schildhauer, Charlotte Cibura

**Affiliations:** 1 Department of General and Trauma Surgery, BG University Hospital Bergmannsheil Bochum, Ruhr University Bochum, Bürkle-de-la-Camp-Platz 1, 44789 Bochum, Germany; 2 Department of Spinal Cord Injuries, BG University Hospital Bergmannsheil, Ruhr University Bochum, Bochum, Germany

**Keywords:** Single-joint hybrid assistive limb, Rehabilitation, Total knee arthroplasty, Knee function

## Abstract

**Introduction:**

The first weeks after total knee arthroplasty (TKA) are crucial for the functional outcome. To improve knee mobility, a continuous passive motion (CPM) motor rail is commonly used during in-hospital rehabilitation. The single-joint hybrid assistive limb (HAL-SJ) is a new therapy device. The aim of the study was to improve patients’ range of motion (ROM), mobility, and satisfaction using the active-assistive support of the HAL-SJ.

**Materials and methods:**

Between 09/2017 and 10/2020, 34 patients, who underwent TKA and matched the inclusion criteria, were randomized into study (HAL-SJ) and control (CPM) group. Treatment began after drain removal and was carried out until discharge. Primary outcome parameters were raised pre- and postoperatively and included the Oxford knee score (OKS), visual analog scale (VAS), and acquired range of motion. Furthermore complications caused by the device were recorded.

**Results:**

OKS increased in both groups postoperatively, but only significantly in the HAL-SJ group. Postoperative pain improved in both groups without significant differences. Flexion improvement was significant in both groups between days 3/7 and 8 weeks postoperatively. We did not encounter any complications related to HAL-SJ.

**Conclusions:**

In conclusion, use of the HAL-SJ during rehabilitation in the early postoperative period after TKA was safe without disadvantages compared to the control group and seems to have advantages in terms of daily life impairment.

## Introduction

After total knee arthroplasty (TKA), rehabilitation during the early postoperative period is decisive for the functional outcome. Previous studies showed a decrease in knee mobility in the first month after TKA due to pain and dysfunction of the M. quadriceps femoris [1]. To improve the range of motion (ROM) and the mobility, a continuous passive motion (CPM) motor rail is commonly used to assist the passive movements of the affected knee joint in early postoperative care. Former studies could not show a uniform, significant improvement when CPM was used postoperatively [2–4], so that there is still a lot of controversy and discussion on CPM use after TKA. Therefore, it is desirable to find a new tool, which can be used for rehabilitation after TKA.

The single-joint hybrid assistive limb (HAL-SJ, Cyberdyne Inc., Tsukuba, Japan) is a recently developed, neurologically controlled therapy device supporting flexion and extension of the knee joint triggered by patient’s own bioelectrical signals (BES) [5, 6]. It was developed as single-joint version of the robot suit HAL. In contrast to other exoskeletons, which mainly work posture-controlled and hereby generate a direct motion support [7], the hybrid assistive limb (HAL) is voluntarily and neurologically controlled by the patient’s own bioelectrical muscle signals [8, 9].

Recent publications revealed a somatosensory feedback-loop that enhances neural plasticity and locomotor learning after spinal cord injury which significantly improves walking ability [8, 10, 11]. The effectiveness of training with robot suit HAL in patients suffering from a spinal cord injury has already been demonstrated in several studies [5, 8]. Moreover, several studies showed the effectiveness of the HAL system for patients suffering from paralyzed limbs after a stroke [12–14]. In case of hemiplegia a single leg version can be used [15]. Also in cardiac rehabilitation for patients with chronic heart failure first promising results have been published [16].

Several pilot and case studies investigated the use of the HAL-SJ in the rehabilitation after knee and elbow injuries, but of these only a few studies analysed the effects after TKA [17–23]. While the HAL-SJ may shorten the rehabilitation regarding time and effort (by improving mobility and agility more quickly), there are no insights regarding a possible improvement of the functional outcome especially with regard to activities of daily living yet.

This study aims to demonstrate safe application of the HAL-SJ in patients after TKA and secondly addresses possible improvements in ROM, patients’ satisfaction, and mobility.

## Materials and methods

This is a clinical, prospective pilot study for application of HAL-SJ as a new therapy device, which was approved and authorized by the Institutional Review Board (Register Number 16-5979). Patients who underwent TKA due to osteoarthritis (degenerative, posttraumatic) between 09/2017 and 10/2020 were included. Strict exclusion criteria were applied: age older than 70 years, Kellgren–Lawrence score < °2, American Society of Anesthesiologists (ASA) score > III, paralysis of the lower extremity, Parkinson's disease, muscular dystrophies, skin diseases aggravated by the application of electrodes, massive edema of the lower extremity with a maximum thigh circumference > 70 cm, body mass index > 40 kg/m^2^, pain that makes a conventional rehabilitation impossible, non-device related revision surgery, rejection by patient. Furthermore patients depending on cardiac pacemakers were excluded due to possible interactions with the device. Patients were randomly divided into two groups by a randomized list. Surgery was performed in a conventional manner by several main surgeons at an endoprosthetics center of maximum care (EndoCert certified hospital). Different TKA implant systems were used (Table 1). Patient demographics and general information were collected from patients’ clinical record. For clinical assessment Oxford knee score (OKS) was assessed preoperatively (day − 1) and at follow-up, approximately 8 weeks postoperatively. The OKS is a questionnaire consisting of 12 questions. Up to 48 points (4 for each question) can be achieved as best of all result. Based on the questionnaire, the function and pain of the affected knee joint and the resulting daily life impairment can be assessed. In addition pain by visual analog scale (VAS) and measurement of the passive knee joint ROM were assessed preoperatively (day − 1), on days 3, 7 and 8 weeks postoperatively. For VAS the patient had to mark the location of his pain on a 100 mm long line, with the worst possible pain in the extreme right (100 mm) and no pain on the left (0 mm). ROM is stated for extension and flexion in degrees. The determination was carried out with the aid of a goniometer.

**Table 1:** Patient demographics and general information

	CPM	HAL-SJ
Age (years)	59.31 ± 6.89	58.13 ± 5.72
Sex		
Male	8	9
Female	8	6
Affected side		
Left	7	8
Right	9	7
Length of hospital stay (days)	9.44 ± 2.63	9.27 ± 3.58
ASA-score	2.00 ± 0.52	1.87 ± 0.64
Kellgren–Lawrence score	3.25 ± 0.45	3.0 ± 0
TKA implant system		
Zimmer NexGen LCCK	4	4
Zimmer NexGen LPS	6	5
Zimmer RHK	0	1
DePuy Attune (PS)	6	4
Implantcast	0	1

*CPM* continuous passive motion, *HAL-SJ* single-joint hybrid assistive limb, *ASA* American Society of Anesthesiologists, *TKA* total knee arthroplasty, *LCCK* legacy constrained condylar knee, *LPS* legacy knee posterior stabilized, *RHK* rotating hinge knee, *PS* posterior stabilized

General physiotherapy, manual therapy and gait training were performed equally in all patients regardless of the treatment group. Device-training started in both groups after removal of drains and was continued twice a day until discharge. Overall, the patients received a comparable number of training sessions with a mean of 10.63 (± 5.20) sessions in the CPM and 10.53 (± 7.15) training sessions with the HAL-SJ.

**Fig. 1:**
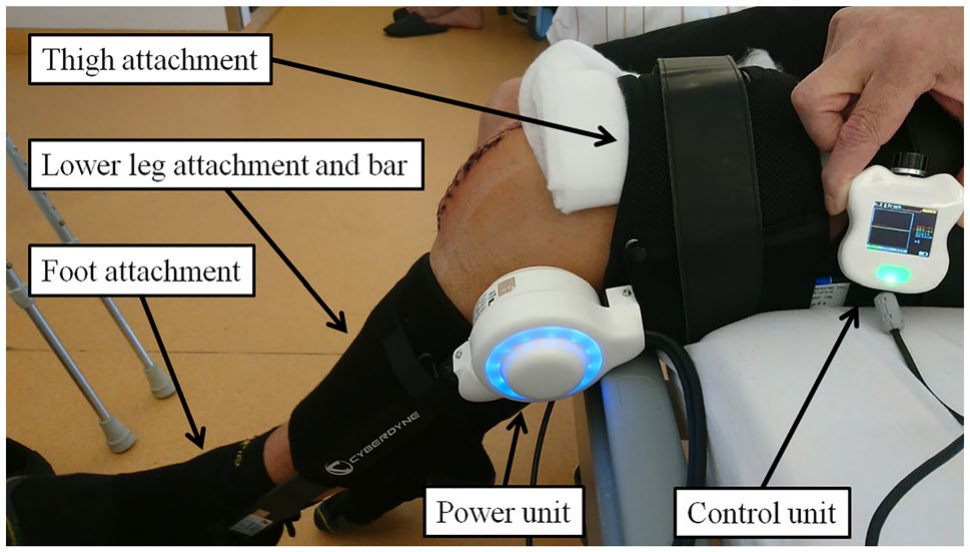
Patient during exercise with HAL-SJ attached to the operated knee after TKA

The HAL-SJ was attached to the patients’ leg and coupled via an electric motor. The BES of the M. vastus lateralis and M. biceps femoris were detected via electrodes. Electrodes were placed according to the SENIAM guideline [24]. In the cybernic voluntary control mode, the patient was then supported BES triggered in extension and flexion in the knee joint. The patient sits at a bed edge and performs the extension and flexion in the knee joint for the affected knee (Fig. 1). The extent of movement is controlled by the patient himself. Five sets with 20 repetitions were performed twice a day (rest between sets 5 min, total duration approximately 30–45 min per unit).

In the control group, rehabilitation of the patients took place using CPM motor rail. In this case, the patient was lying in his bed and his leg was placed and fixed in a motor rail. The knee joint was passively moved, whereby the amount of movement for the flexion was initially started at 60°. The flexion was increased as soon as the patient was able to perform more and was raised up to 90° during the in-hospital stay. Treatment with the motor rail was carried out twice a day for 30–45 min.

We assessed complications caused by the HAL-SJ in terms of possible wound healing, soft tissue injuries like muscle fibre tears and severe complications like periprosthetic fractures or implant loosening. We assessed routinely taken pre-discharge radiographs to evaluate a possible loosening and/or fracture.

Statistical analyses were performed using GraphPad Prism (version 7.05, GraphPad Software Inc., CA, USA). Data are given as mean ± standard deviation (SD). Significances between the mean values of the study group and the control group or in the comparison between the study times within a group have been calculated using unpaired and paired student’s *t* test after verification normality of the distribution of the data. Results were considered statistically significant when the *p* value was < 0.05 and highly significant < 0.01.

## Results

Thirty-four patients who matched the inclusion criteria were included in the trial between 09/2017 and 10/2020. Three patients (two in the study group, one in the control group) dropped out because of (1) incompliance, (2) postoperative, non-device related infection (subcutaneous abscess lower leg, unrelated to TKA), (3) wound revision surgery due to a postoperative fall on the ward. Therefore, a total of 31 patients (*n* = 15 study group and *n* = 16 control group) were taken into account for the final evaluation.

The demographic and general data of the patients were comparable in both groups. Data are shown in Table 1.

We did not encounter any complications related to HAL-SJ. Striking was the fact that a continuous femoral nerve block with high local anaesthetic dose used for postoperative pain management made it impossible for the patient to develop sufficient BES for HAL-SJ training.

### Oxford knee score

The OKS showed a mean of 19.94 (± 5.77) points in the control group preoperatively. That indicates moderate to severe knee arthritis [25].

**Fig. 2:**
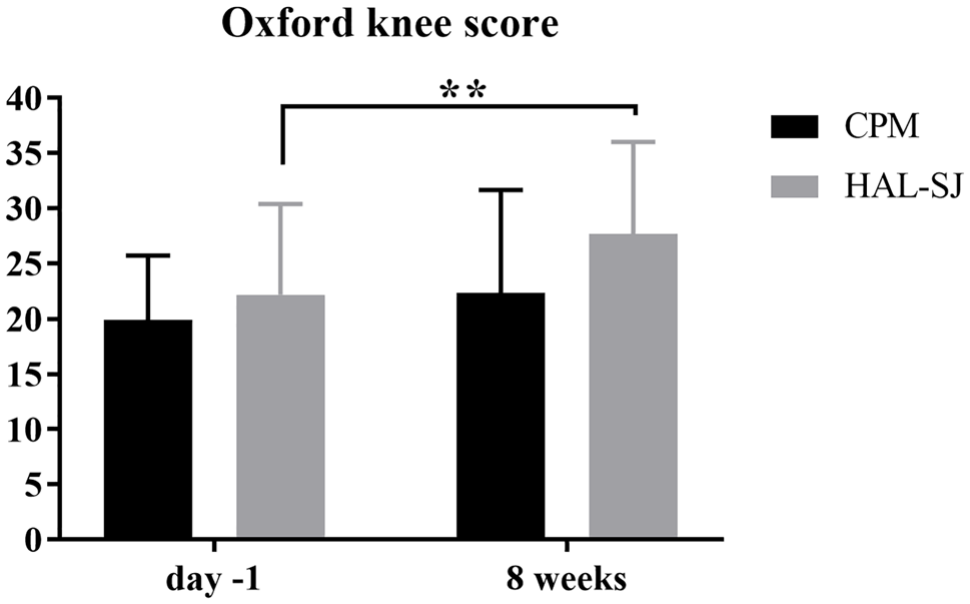
Mean value and standard deviation of the Oxford knee score (in points) on day − 1 (preoperative) and 8 weeks postoperative for control group (CPM, continuous passive motion) and study group (HAL-SJ, single-joint hybrid assistive limb). There was a highly significant (**p < 0.01) improvement in the HAL-SJ group

The score increased to 22.31 (± 9.33, *p* = 0.09) in the postoperative survey, that was assessed in the routine follow-up examination approximately 8 weeks after surgery (59.94 ± 24.94 days). In the HAL-SJ group, the mean of OKS showed a highly significant increase (*p* = 0.003) from 22.13 (± 8.24) preoperative to 27.67 (± 8.35) points at 8 weeks postoperative (Fig. 2, Table 2). Differences between the two groups were not significant (day − 1 *p* = 0.39, 8 weeks *p* = 0.10).

**Table 2:** Summary of results

	CPM				HAL-SJ			
	Day − 1	Day 3	Day 7	8 weeks	Day − 1	Day 3	Day 7	8 weeks
OKS (points)								
Mean	19.94	–	–	22.31	22.13	–	–	27.67
SD	5.77	–	–	9.33	8.24	–	–	8.35
VAS improvement (mm)								
Mean	–	16.25	19.08	27.25	–	10.53	21.5	20.34
SD	–	27.79	19.10	31.86	–	22.06	26.09	29.80
Extension deficit (°)								
Mean	− 1.25	− 1.88	− 1.00	− 0.81	− 1.67	− 0.67	− 0.83	− 1.67
SD	2.24	3.10	2.83	1.80	3.09	1.76	1.95	2.44
Flexion (°)								
Mean	113.13	72.19	78.85	102.81	110.67	79.00	82.92	105.33
SD	15.80	17.12	18.16	12.11	15.68	12.71	15.88	13.43

*CPM* continuous passive motion, *HAL-SJ* single-joint hybrid assistive limb, *OKS* Oxford knee score, *VAS* visual analog scale for pain, *SD* standard deviation

### Visual analog scale (VAS) for pain

The pain assessment by means of VAS showed an improvement in pain postoperative in both groups. Compared to the day before surgery, on day 3 postoperative, it decreased for HAL-SJ group by 10.53 mm (± 22.06 mm) and 16.25 mm (± 27.79 mm) for CPM group. Day 7 showed an improvement on the VAS by 21.5 mm (HAL-SJ, ± 26.09 mm), respectively, 19.08 mm (CPM, ± 19.10 mm). At the point of final follow-up after 8 weeks compared to preoperative data, we measured an improvement of 20.34 mm (± 29.80 mm) for the HAL-SJ group and 27.25 mm (± 31.86 mm) for the control group. There were no significant differences between the groups. A summary of results, including the improvement of VAS between day − 1 and the respective measurement day, is shown in Table 2.

### Range of motion

Passive ROM showed no significant changes for the extension. Extension deficit was 1.67 (± 3.09), 0.67 (± 1.76), 0.83 (± 1.95), and 1.67 (± 2.44) degrees for HAL-SJ group on day − 1, 3, 7, and 8 weeks postoperative. In the CPM group passive extension deficit was 1.25 (± 2.24), 1.88 (± 3.10), 1.00 (± 2.83), and 0.81 (± 1.80) degrees (Table 2).

**Fig. 3:**
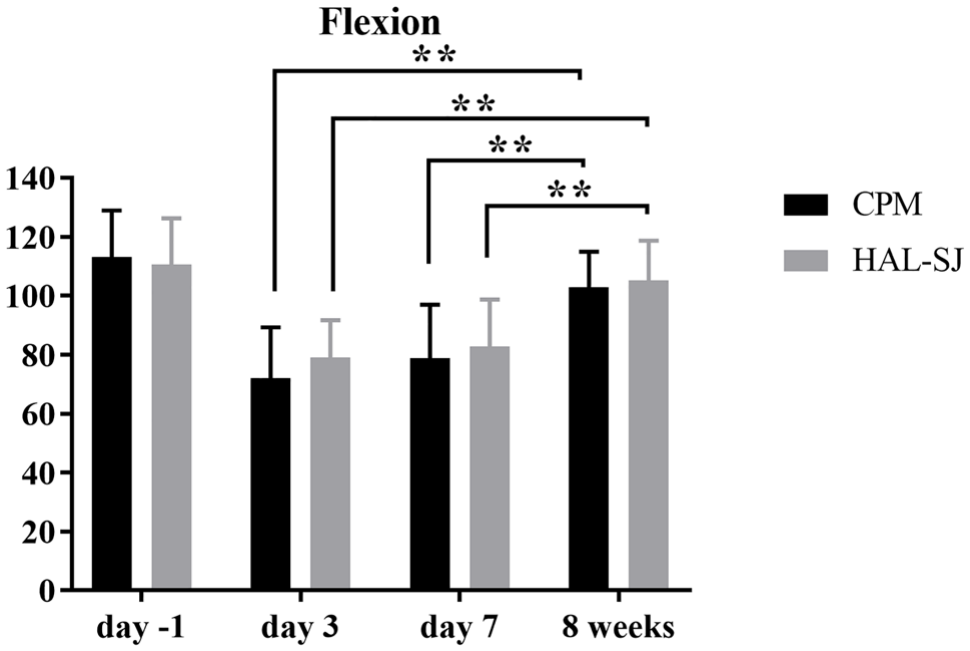
Mean values and standard deviation for passive knee flexion on day − 1 (preoperative) as well as day 3, day 7, and 8 weeks postoperative in the control group (CPM, continuous passive motion) and study group (HAL-SJ, single-joint hybrid assistive limb). In both groups there was a highly significant (**p < 0.01) improvement of flexion both between day 3 and 8 weeks and between day 7 and 8 weeks

On the measurement days the mean values of flexion in the HAL-SJ group were 110.67 (± 15.68), 79.00 (± 12.71), 82.92 (± 15.88), and 105.33 (± 13.43) degrees and in the CPM group 113.13 (± 15.80), 72.19 (± 17.12), 78.85 (± 18.16), and 102.81 (± 12.11) degrees (Table 2). The determination of flexion showed in both groups a highly significant (*p* < 0.01) improvement in flexion between days 3/7 and 8 weeks (HAL-SJ day 3 to 8 weeks *p* < 0.001, day 7 to 8 weeks *p* = 0.004, CPM day 3 to 8 weeks *p* < 0.001, day 7 to 8 weeks *p* < 0.001). Differences between the groups were not significant. Results are shown in Fig. 3.

## Discussion

Rehabilitation after TKA is an important challenge due to the high and during the last years' constantly increasing number of patients undergoing TKA [26]. Especially for younger patients suffering from advanced knee arthritis, aim is to increase the functional outcome and daily life activity or even the ability to return to job or sports. Apart from conventional physiotherapy, therapy devices could help to improve and maybe accelerate the rehabilitation program with benefits for the patient and society/health care system (cost savings). Therefore, first aim of the study was to demonstrate safe application of the HAL-SJ in patients after TKA. No adverse events occurred in our study group. While this confirms other studies, which could also demonstrate a safe application [17, 23, 27], certain limitations associated with the HAL-SJ-use appeared.

In terms of patient demographics and general information there were no significant differences between the two groups. Regarding the time period of the study the number of included patients is small, primarily due to the extensive exclusion criteria. We had a drop out of three patients. One patient in the HAL-SJ group dropped out because of incompliance in the training sessions with the HAL-SJ. Cooperation of the patient is necessary to use the active-assistive support of the new therapy device, while the CPM motor rail is less dependent on the patients’ collaboration. Therefore, patient education is fundamental using HAL-SJ.

Another patient of the HAL-SJ group dropped out because of a postoperative subcutaneous abscess on the lower leg (unrelated to TKA). This was clearly not related to the device-use. Especially no wound dehiscence occurred during training sessions. In comparison, there was one drop out in the control group as well. Due to a postoperative fall on the ward, independent from device-training sessions, wound revision surgery needed to be performed.

Despite the safe application of the HAL use, we noticed difficulties, if a continuous femoral nerve block was used for postoperative pain management. Quadriceps weakness, which can also lead to a higher rate of falls, is a known complication of using femoral nerve blocks [28, 29]. We suggest preferring other types of pain therapy when using HAL-SJ as therapy device. But also use of femoral nerve blocks is discussed controversy, not being able to administer it after TKA is a shortcoming of HAL-SJ in comparison to CPM. Another possibility is to start training with the HAL-SJ later after surgery. However, this study showed that the time of training might be early as we started training with both therapy devices (HAL-SJ and CPM) as soon as the drain was removed after surgery. Compared to recently published studies we hereby increased the training intensity with the HAL device in a real clinical setting of an endoprosthetics-center of maximum care. As training was performed twice a day, a good number of training sessions was performed by each patient until discharge. Previous studies started HAL-SJ exercises on day 5 [17, 27] or even day 10 [23], but only described few numbers of training sessions. Still optimal number and time of training sessions with HAL-SJ to achieve maximal effectiveness needs to be found as this was not aim of the study.

We suggested that the active-assistive support of the HAL-SJ could improve patients’ range of motion (ROM), mobility and satisfaction. As support of the HAL is self-initiated by muscle activity (through BES) of the patient, a higher training effect compared to only passive movement is suggested. Therefore, this is the first study which shows an improvement of daily life impairment after TKA with HAL-SJ training. OKS hereby showed a highly significant postoperative improvement, while the improvement was not significant in the control group. Nevertheless, the postoperative differences between the two groups were not significant. Kotani et al. used the EQ5D5L score as quality of life assessment 6 month after TKA [27]. They also showed a better value for HAL-SJ group but without comparing it to preoperative values [27].

Pain is an important parameter for evaluation of the outcome after TKA as higher pain levels lead to dissatisfaction of the patients and difficulties in mobilization [30]. Former studies showed that VAS is a good tool to assess the severity of pain [31]. In our study, we could demonstrate that although training is performed with active-assistive support pain assessment by VAS was comparable in both groups. However, we have not documented pain management for each patient individually in our study design. That makes the interpretation of data difficult. Pain decreased during follow-up in both groups without significant differences between groups. This confirms the results published by Yoshioka et al. who showed a slight but not significant reduction of pain following the HAL-SJ intervention [23]. Goto et al. and Kotani et al. measured pain levels through VAS during active knee movement and compared that to VAS during either HAL-SJ assisted or conventional active-assistive exercises. A significant larger degree of improvement for changes in the VAS was found in the HAL-SJ group [17, 27].

ROM, as an important indicator of successful TKA [32], was assessed preoperatively as well as on days 3, 7 and 8 weeks postoperatively. After surgery the ROM decreases. Aim of rehabilitation is then to increase the ROM due to physiotherapy and training. We could demonstrate that with both CPM and HAL-SJ training a significant improvement up to the point of final follow-up after 8 weeks was achieved. Nevertheless, we could not assess a significant higher improvement under HAL-SJ training. Kotani et al. assessed active and passive ROM on postoperative days 5 and 10 and could demonstrate a significant improvement in the HAL-SJ compared to the control group, while there was also no significant difference between the groups at the 6-month follow-up [27]. Possible explanations for the significant improvement under HAL-SJ training shown by Kotani et al. can be the different study design (CPM used in both study groups, HAL-SJ training is performed additionally, active exercise is performed in the control group), the different training points starting on day 5 after surgery and the different follow-up period (baseline: day 5, post-treatment evaluation: day 10).Other studies focused on the extension lag without measuring full ROM [17, 23]. Regarding ROM, advantage of the HAL-SJ is to provide higher patient-control as movement range and support of the device is dependent on the patients’ own endpoints. In contrast, CPM only works in the range that is set by the therapist. The use of the CPM motor rail as well as the use of the HAL-SJ could also save human resources. Similar results have been shown in spinal cord injuries with use of the robot suit HAL by reducing the demand and workload for the physiotherapists [10]. Additionally, HAL-SJ can be easily transported in a small case and is convenient to use. However, it is currently not yet available for standard use. Therefore, at this moment, the findings of the study can only be transferred in a limited extent to everyday clinical practice.

The study has limitations. Due to the study design testing a new therapy device strict exclusion criteria were applied. Hereby only young and healthy patients were included (compared to average person receiving TKA). There was also more than one main surgeon and different TKA implant systems were used (almost balanced between the groups). Our follow-up period is short so that other studies with longer follow-up periods are necessary to measure long-term results. Furthermore, due to patient discharge after about 9 days and only possible in-hospital training with HAL-SJ, the training period was limited. As mentioned before, optimal time and number of training sessions need to be found. Additionally larger study groups are necessary to achieve more significant results. Other parameters to determine muscle strength and patients’ satisfaction are desirable.

## Conclusions

The use of the HAL-SJ during rehabilitation in the early postoperative period after TKA showed good characteristics in terms of feasibility, safety, and patient acceptance. Results showed no disadvantages compared with the control group. The expected significantly better results for range of motion could not be shown in this setting. However, OKS showed a highly significant postoperative improvement for the HAL-SJ group. Therefore, in terms of daily life impairment, the use of the HAL-SJ seems to have advantages. Larger study groups and a longer treatment period with the device are necessary in future studies.

## Abbreviations

TKA: Total knee arthroplasty
CPM: Continuous passive motion
HAL-SJ: Single-joint hybrid assistive limb
ROM: Range of motion
OKS: Oxford knee score
VAS: Visual analog scale
BES: Bioelectrical signals
ASA: American Society of Anesthesiologists
SD: Standard deviation

## References

[1] Mizner RL, Petterson SC, Snyder-Mackler L (2005). Quadriceps strength and the time course of functional recovery after total knee arthroplasty. J Orthop Sports Phys Ther.

[2] Boese CK, Weis M, Phillips T (2014). The efficacy of continuous passive motion after total knee arthroplasty: a comparison of three protocols. J Arthroplasty.

[3] Harvey LA, Brosseau L, Herbert RD (2014). Continuous passive motion following total knee arthroplasty in people with arthritis. Cochrane Database Syst Rev.

[4] Liao C-D, Huang Y-C, Lin L-F (2016). Continuous passive motion and its effects on knee flexion after total knee arthroplasty in patients with knee osteoarthritis. Knee Surg Sports Traumatol Arthrosc.

[5] Aach M, Cruciger O, Sczesny-Kaiser M (2014). Voluntary driven exoskeleton as a new tool for rehabilitation in chronic spinal cord injury: a pilot study. Spine J.

[6] Fisahn C, Aach M, Jansen O (2016). The effectiveness and safety of exoskeletons as assistive and rehabilitation devices in the treatment of neurologic gait disorders in patients with spinal cord injury: a systematic review. Global Spine J.

[7] Aach M, Meindl RC, Geßmann J (2015). Exoskelette in der Rehabilitation Querschnittgelähmter. Möglichkeiten und Grenzen (Exoskeletons for rehabilitation of patients with spinal cord injuries. Options and limitations). Unfallchirurg.

[8] Sczesny-Kaiser M, Höffken O, Aach M (2015). HAL® exoskeleton training improves walking parameters and normalizes cortical excitability in primary somatosensory cortex in spinal cord injury patients. J Neuroeng Rehabil.

[9] Sczesny-Kaiser M, Trost R, Aach M (2019). A randomized and controlled crossover study investigating the improvement of walking and posture functions in chronic stroke patients using HAL exoskeleton—the halestro study (HAL-Exoskeleton STROke Study). Front Neurosci.

[10] Jansen O, Schildhauer TA, Meindl RC (2017). Functional outcome of neurologic-controlled HAL-Exoskeletal neurorehabilitation in chronic spinal cord injury: a pilot with one year treatment and variable treatment frequency. Glob Spine J.

[11] Jansen O, Grasmuecke D, Meindl RC (2018). Hybrid assistive limb exoskeleton HAL in the rehabilitation of chronic spinal cord injury: proof of concept; the results in 21 patients. World Neurosurg.

[12] Hyakutake K, Morishita T, Saita K (2019). Effects of home-based robotic therapy involving the single-joint hybrid assistive limb robotic suit in the chronic phase of stroke: a pilot study. Biomed Res Int.

[13] Oga K, Yozu A, Kume Y (2020). Robotic rehabilitation of the paralyzed upper limb for a stroke patient using the single-joint hybrid assistive limb: a case study assessed by accelerometer on the wrist. J Phys Ther Sci.

[14] Wall A, Borg J, Vreede K (2020). A randomized controlled study incorporating an electromechanical gait machine, the Hybrid Assistive Limb, in gait training of patients with severe limitations in walking in the subacute phase after stroke. PLoS ONE.

[15] Kawamoto H, Hayashi T, Sakurai T (2009). Development of single leg version of HAL for hemiplegia. Annu Int Conf IEEE Eng Med Biol Soc.

[16] Watanabe H, Koike A, Wu L (2019). Efficacy of Cardiac Rehabilitation with Assistance from Hybrid Assistive Limb in Patients with Chronic Heart Failure: Protocol for a Randomized Controlled Study. Cardiology.

[17] Goto K, Morishita T, Kamada S (2017). Feasibility of rehabilitation using the single-joint hybrid assistive limb to facilitate early recovery following total knee arthroplasty: a pilot study. Assist Technol.

[18] Kubota S, Kadone H, Shimizu Y (2018). Robotic rehabilitation training with a newly developed upper limb single-joint Hybrid Assistive Limb (HAL-SJ) for elbow flexor reconstruction after brachial plexus injury: a report of two cases. J Orthop Surg (Hong Kong).

[19] Kubota S, Mutsuzaki H, Yoshikawa K (2019). Safety and efficacy of robotic elbow training using the upper limb single-joint hybrid assistive limb combined with conventional rehabilitation for bilateral obstetric brachial plexus injury with co-contraction: a case report. J Phys Ther Sci.

[20] Saita K, Morishita T, Hyakutake K (2020). Feasibility of robot-assisted rehabilitation in poststroke recovery of upper limb function depending on the severity. Neurol Med Chir (Tokyo).

[21] Yoshioka T, Sugaya H, Kubota S (2016). Knee-extension training with a single-joint hybrid assistive limb during the early postoperative period after total knee arthroplasty in a patient with osteoarthritis. Case Rep Orthop.

[22] Yoshioka T, Kubota S, Sugaya H (2017). Robotic device-assisted knee extension training during the early postoperative period after opening wedge high tibial osteotomy: a case report. J Med Case Rep.

[23] Yoshioka T, Kubota S, Sugaya H (2021). Feasibility and efficacy of knee extension training using a single-joint hybrid assistive limb, versus conventional rehabilitation during the early postoperative period after total knee arthroplasty. J Rural Med.

[24] Hermens HJ (1999) European recommendations for surface ElectroMyoGraphy: results of the SENIAM project. SENIAM, vol 8. In: Roessingh Research and Development, Enschede

[25] Dawson J, Fitzpatrick R, Murray D (1998). Questionnaire on the perceptions of patients about total knee replacement. J Bone Joint Surg Br.

[26] Worlicek M, Koch M, Daniel P (2021). A retrospective analysis of trends in primary knee arthroplasty in Germany from 2008 to 2018. Sci Rep.

[27] Kotani N, Morishita T, Saita K (2020). Feasibility of supplemental robot-assisted knee flexion exercise following total knee arthroplasty. J Back Musculoskelet Rehabil.

[28] Bauer MCR, Pogatzki-Zahn EM, Zahn PK (2014). Regional analgesia techniques for total knee replacement. Curr Opin Anaesthesiol.

[29] Yang X, Kang W, Xiong W (2019). The effect of dexmedetomidine as adjuvant to ropivacaine 0.1% for femoral nerve block on strength of quadriceps muscle in patients undergoing total knee arthroplasty: a double-blinded randomized controlled trial. J Pain Res.

[30] Singelyn FJ, Deyaert M, Joris D (1998). Effects of intravenous patient-controlled analgesia with morphine, continuous epidural analgesia, and continuous three-in-one block on postoperative pain and knee rehabilitation after unilateral total knee arthroplasty. Anesth Analg.

[31] Boeckstyns MEH, Backer M (1989). Reliability and validity of the evaluation of pain in patients with total knee replacement. Pain.

[32] Ritter MA, Campbell ED (1987). Effect of range of motion on the success of a total knee arthroplasty. J Arthroplasty.

